# Technical NEMA NU2–2018 Performance Assessment of Time-of-Flight-Integrated Digital PET-CT System

**DOI:** 10.1055/s-0044-1778709

**Published:** 2024-01-22

**Authors:** Manoj Kumar Singh, Krishan Kant Agarwal, Manish Vishwakarma, Hemant Patel

**Affiliations:** 1 Medikabazaar, Technopolis Knowledge Park, Mumbai, Maharashtra, India; 2 Gujarat Imaging Centre, Ahmedabad, Gujarat, India

**Keywords:** digital PET-CT, NEMA, NEMA NU2–2018, PET, PET-CT, resolution, sensitivity, TOF

## Abstract

**Aim** The objective of this study includes the NEMA (National Electrical Manufacturer Association) NU2–2018 performance evaluation of the uMIvista PET-CT (positron emission tomography-computed tomography) system.

**Methods** The latest NEMA NU2–2018 guidelines have been followed for the evaluation of performance parameters of this PET-CT scanner: axial, tangential, and radial spatial resolution, sensitivity, counting losses, scatter, randomness, random and counting loss correction, image quality, time and energy resolution, image uniformity, and image registration alignment post installation of country first uMIvista PET-CT.

**Results** The measured NEMA sensitivity of the uMIvista PET scanner was 12.053 cps/kBq. The spatial resolutions of the PET were measured as tangential, radial, and transaxial spatial resolutions at 10 mm, with 3.01 mm, 2.95 mm, and 2.93 mm, respectively; at 100 mm, with 3.17 mm, 3.42 mm, and 3.05 mm, respectively; and at 200 mm, with 3.65 mm, 4.54 mm, and 3.17 mm, respectively, at full-width half-maximum (FWHM); while at full-width tenths-maximum (FWTM), the values at 10 mm were 5.79 mm, 5.57 mm, and 5.69 mm, respectively, and at 100 mm were 5.59 mm, 5.96 mm, and 5.91 mm, respectively. The measured time-of-flight (TOF) timing resolution was 302.294 ps and the measured energy resolution was 11.76% with FWHM and FWTM.

**Conclusion** The NEMA NU2–2018 performances of this TOF-integrated digital PET-CT system are extremely good in all parameters.

## Introduction

The positron emission tomography-computed tomography (PET-CT) scanner is used as a diagnostic imaging tool in the treatment of oncological and nononcological diseases. 1 The special feature is that it is a hybrid fusion modality. The PET component of this modality plays a crucial role in functional imaging to measure the physiology and metabolism of tumors in the human body. 2 The CT portion offers dual functionality in PET-CT imaging: one as a standard diagnostic CT to provide diagnostic and anatomical information similar to the standalone diagnostic CT scanner, and the other as a PET-assisted modality to provide the CT data for the attenuation corrections of PET images. 3 Attenuation correction CT data can be diagnostic and nondiagnostic. The nondiagnostic CT data are acquired with low-radiation exposure factors (low milliampere seconds [mAs] and peak kilovolt [kVp]). Technological advances in PET-CT, such as improvements in image quality, performance parameters, and the introduction of deep learning reconstructions in PET, are leading to major changes in nuclear medicine practice and patient diagnosis management. 4 5 The recent introduction of digital PET-CT technology has led to a reduction in absorbed radiation dose to patients by PET imaging due to a reduction in injection of radionuclides as well as a reduction in radiation dose by CT imaging of PET-CT. 4 6 The digital PET-CT systems are integrated with a semiconductor-based silicon photomultiplier (SiPM). It is a replacement for the photomultiplier glass tube (PMT). At present, most of the PET-CT manufacturers have already released their digital PET-CT systems and only a few of them have replaced their traditional analog PET-CT system with a digital PET-CT. Our facility recently installed the United Imaging Healthcare uMIvista PET-CT system. This is the country's one of the initial uMIvista digital PET-CT system. uMIvista has a 24 cm wide PET axial field of view (AFOV) with integrated digital SiPM detector, lutetium yttrium oxyorthosilicate (LYSO) crystal with time-of-flight (TOF) technology, and 160-slice CT scanner with the fastest rotation time of 0.3 second. 4 7

This study includes the National Electrical Manufacturer Association (NEMA) NU2–2018 performance evaluation of the uMIvista PET-CT system. The latest NEMA NU2–2018 guidelines have been followed for the evaluation performance parameters of this PET-CT scanner: axial, tangential, and radial spatial resolution, sensitivity, counting losses, scatter, randomness, random and counting loss correction, image quality, time and energy resolution, image uniformity, and image registration alignment. All assay performance tests were performed using the radiopharmaceuticals fluoro-18-sodium fluoride and 18F-fluorodeoxyglucose (18F-FDG). 4 7 9 10 11 12

## Methods

### uMIvista PET-CT System

The uMIvista PET-CT (Shanghai United Imaging Healthcare China) system is integrated with LYSO crystals (Lu1.8Y.2SiO5:Ce) with SiPM detectors and AFOVs of 24 cm. This PET-CT system is equipped with an 80-row, 160-slice CT scanner. The width of the CT detector is 40 mm. As for the PET hardware, the system is integrated into a 64680 LYSO crystal connected to SiPM detector modules. The crystal size is 2.76 × 2.76. The time window is 4 nanoseconds and the system is integrated with three-dimensional TOF-mode acquisition.

### Technical Performance Parameter Assessment: Spatial Resolution and Sensitivity, Count Losses, Scatter, Randoms Fraction, Correction of Random and Counts Loss, Image Quality, Timing and Energy Resolution, Image Uniformity, and Image Registration Alignment

The NEMA technical performance of the integrated TOF-PET digital system was calculated using the NEMA NU2–2018 guidelines standard. 4 7 8 9 The NEMA parameter evaluation of the PET-CT system included spatial resolution (axial, radial, and tangential at 10, 100, and 200 mm), NEMA sensitivity, NEMA image quality, contrast recovery coefficient (CRC) and background variability (BV), image uniformity, count loss correction, scatter fraction accuracy, TOF resolution, count accuracy, count rate performance (noise-equivalent count rate [NECR]), single and coincidence count corrections, dead time correction, image uniformity, and PET-CT image registration. According to NEMA NU2–2018 guidelines, axial, radial, and tangential spatial resolutions were measured using an 18F-FDG point source. 4 7 11 12 The measurements of the spatial resolution were performed at 10, 100, and 200 mm from the center. The 18F-FDG point source was manufactured in the radiopharmaceutical laboratory of a PET-CT facility and has a radioactivity concentration of approximately 1 Megabecquerel (MBq) as specified in the PET-CT manufacturer's instructions for use. The source was positioned on the scanner and data collection was performed according to the NEMA protocol. Axial, tangential, and radial spatial resolutions were measured at 1/2 and 1/8 AFOV of the PET, and the average resolution was calculated. The NEMA sensitivity test was performed using a sensitivity phantom. The phantom was prepared with 18F-FDG solution in the radiopharmaceutical laboratory behind the lead (Pb)-shielded L bench by filling the phantom tubing with 18F-FDG radiopharmaceutical. Activity was approximately 20 MBq during scan acquisition. The phantom was scanned at the center and 100 mm from the AFOV. Scans were captured according to the scanner's default protocol. A coincidence window was used to correct for random coincidence events. A NEMA phantom with a length of 700 mm and a diameter of 200 mm was set up to assess the scatter fraction, the count losses, and the random fraction. The phantom was prepared and filled with approximately 450 MBq 18F-FDG. The prepared phantom was located isocentrically on the PET-CT scan table and the scan was recorded. This was a long scan of around 12 hours that was done overnight. The collected data were processed according to NEMA NU2–2018 to calculate the performance values. The acquired raw NEMA-PET data were used by retrospective reconstruction, and counting losses and random corrections were formulated. The Monte Carlo method and coincidence time window were used to assess scatter fraction, random fraction, and count loss. The standard protocol was followed with TOF, expectation maximization of ordered subsets, and point spread function reconstruction. A NEMA phantom was prepared and used to calculate the image quality of the acquired PET images. The phantom had spherical spaces with different diameters (10, 13, 17, 22, 28, and 37 mm); these were prepared with 18F-FDG radioactivity. The rest of the Phantom had a capacity of 9.8 L. The radioactivity of the 18F-FDG concentration was four times the background volume of the phantom. The radioactivity of the bead was approximately 120 MBq during PET data acquisition. The total scan time was approximately 30 minutes, with the standard scan time being 5 minutes per image. The CT scan was also acquired for attenuation correction of PET images. The captured images were used to evaluate image quality. CRC, BV, and relative lung error were calculated. TOF resolution and energy resolution were calculated using full-width half-maximum (FWHM) and full-width tenths-maximum (FWTM).

## Results

### Spatial Resolution and Sensitivity

**Fig. 1:**
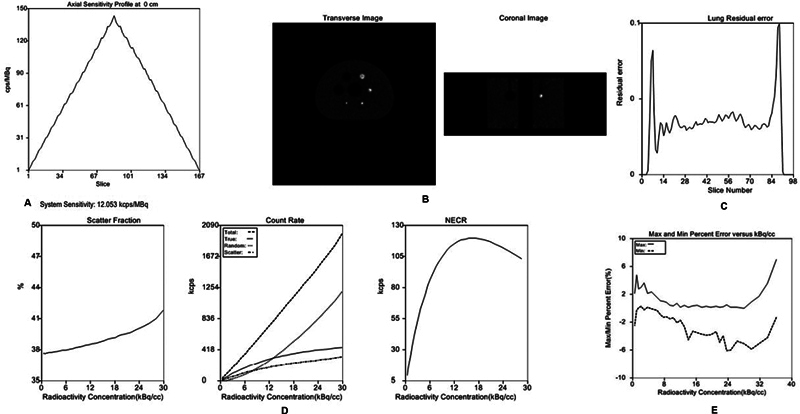
( **A** ) NEMA sensitivity: ( **B** , **C** ) PET image of the image quality phantom, ( **B** ) transverse and coronal image, and ( **C** ) lung relative error; ( **D** ) scatter fraction, counts rate (total, true, random, and scatter), and NECR; and ( **E** ) percentage error versus radioactivity concentration. NECR, noise-equivalent count rate; NEMA, National Electrical Manufacturer Association; PET, positron emission tomography.

The spatial resolutions of the PET were measured as tangential, radial, and transaxial spatial resolutions at 10 mm with 3.01 mm, 2.95 mm, and 2.93 mm, respectively; at 100 mm with 3.17 mm, 3.42 mm, and 3.05 mm, respectively; and at 200 mm with 3.65 mm, 4.54 mm, and 3.17 mm, respectively, at FWHM; while at FWTM, the values at 10 mm were 5.79 mm, 5.57 mm, and 5.69 mm, respectively; at 100 mm were 5.59 mm, 5.96 mm, and 5.91, respectively; and at 200 mm were 6.50 mm, 8.07 mm, and 5.95 mm, respectively. All values are given in Table 1 . The measured NEMA sensitivity of the uMIvista PET scanner was 12.053 cps/kBq in the middle. Fig. 1A shows center axial sensitivity profiles. Axial, tangential, and radial spatial resolutions were measured at ½ and ⅛ AFOV of the PET. Both are summarized in Table 2 and 3 .

**Table 1:** Average resolution over both axial positions

Source location	(0, 10) mm			(0, 100) mm			(0, 200) mm		
Direction	Tangential	Radial	Axial	Tangential	Radial	Axial	Tangential	Radial	Axial
FWHM (mm)	3.01	2.95	2.93	3.17	3.42	3.05	3.65	4.54	3.17
FWTM (mm)	5.79	5.57	5.69	5.59	5.96	5.91	6.50	8.07	5.95

Abbreviations: FWHM, full-width half-maximum; FWTM, full-width tenth-maximum.

**Table 2:** Axial, radial, and tangential resolution for 1/2 AFOV

Source location	(0, 10) mm			(0, 100) mm			(0, 200) mm		
Direction	Tangential	Radial	Axial	Tangential	Radial	Axial	Tangential	Radial	Axial
FWHM (mm)	3.01	3.00	3.02	3.17	3.40	3.01	3.68	4.68	3.17
FWTM (mm)	5.81	5.46	5.71	5.56	5.98	5.81	5.94	8.06	5.93

Abbreviations: AFOV, axial field of view; FWHM, full-width half-maximum; FWTM, full-width tenth-maximum.

**Table 3:** Axial, radial, and tangential resolution for 1/8th AFOV

Source location	(0, 10) mm			(0, 100) mm			(0, 200) mm		
Direction	Tangential	Radial	Axial	Tangential	Radial	Axial	Tangential	Radial	Axial
FWHM (mm)	3.01	2.95	2.93	3.17	3.42	3.05	3.65	4.54	3.17
FWTM (mm)	5.79	5.57	5.69	5.59	5.96	5.91	6.50	8.07	5.95

Abbreviations: AFOV, axial field of view; FWHM, full-width half-maximum; FWTM, full-width tenth-maximum.

### Count Losses, Scatter, Randoms Fraction, and Correction of Random and Counts Loss

**Fig. 2:**
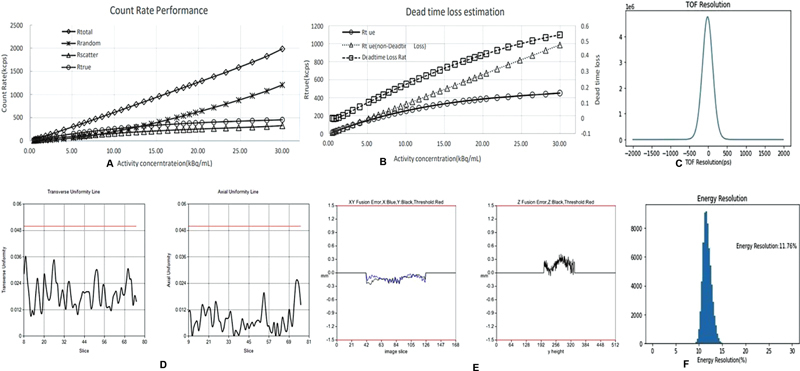
( **A** ) Counts rate performance; ( **B** ) dead time loss estimation; ( **C** ) TOF timing resolution; ( **D** ) image uniformity; ( **E** ) PET-CT image registration alignment; and ( **F** ) energy resolution. PET-CT, positron emission tomography–computed tomography; TOF, time-of-flight.

The measured scatter fraction was 39.15% at a maximum NECR of 452.98 kcps at 29.95 kBq/cc and 37.66% at low activity of 119.95 kcps at 17.42 kBq /cc. The measured peak coincidence count rate is 1,984 kcps and the single peak count rate is 64,495.73 kcps. All values are described in Table 5 and Figs. 1D and 2A,B . The count rate errors at different activity concentrations are shown in Fig. 1E . The maximum percent error below the peak NECR activity at a peak NECR of 119.95 kcps at 17.42 kBq/cc was 4.8%.

**Table 4:** Summary of all contrast recovery coefficients and corresponding background variability

Sphere diameter (mm)	10	13	17	22	28	37
Hot contrast (%)	54.6	60.6	66.5	74.7		
Cold contrast (%)					85.7	88.5
Background variability (%)	6.2	5.1	4.0	3.2	2.7	2.3
Mean lung relative error (%)	2.9					

**Table 5:** Analysis report for scatter fraction, and count rate and loss performance

System scatter fraction @ peak NECR	39.15%
System scatter fraction @ low activity	37.66%
Peak true rate	452.98 (kcps) @ 29.95 (kBq/cc)
Peak NECR	119.95 (kcps) @17.42 (kBq/cc)
Peak coincidence count rate (kcps)	1,984.00
Peak single count rate (kcps)	64,495.73
Peak true count rate (kcps)	452.98
The max % error below the NECR peak activity	4.80%
TOF resolution	302.294 ps
Energy resolution	11.76%
Transverse uniformity (0.00–0.05)	0.036
Axial uniformity (0.00–0.05)	0.025
Accuracy in PET-CT registration (–1.5 to 1.5 mm)	
X direction	–0.2510
Y direction	–0.2620
Z direction	0.4209

Abbreviations: kBq/cc, kilobecquerel per cubic centimeter; NECR, noise-equivalent count rate; PET-CT, positron emission tomography-computed tomography; ps, TOF, time-of-flight.

### Image Quality, TOF Timing Resolution, Energy Resolution, Image Uniformity, and PET-CT Image Alignment

The CRC and BV ratio of all measured bullets was 4:1, and the CRCs of the radioactivity-filled hot and cold bullets were 55.6, 60.6, 66.5, and 74.7% at 10, 13, 17, and 22 mm, respectively, and 85.7 and 88.5% at 28 and 37 mm, respectively; while BV was 6.2, 5.1, 4.0, 3.2, 2.7, and 2.3% at 10, 13, 17, 22, 28, and 37 mm, respectively. The measured relative lung error was 2.9% as shown in Fig. 1C. The hot and cold sphere CRC and BV in the 4:1 ratio is summarized in Table 4 . The image section in transverse and coronal views is shown in Fig. 1B . The measured TOF timing resolution was 302.294 ps and the measured energy resolution was 11.76% with FWHM and FWTM. The TOF resolution and energy resolution are summarized in Table 5 and Fig. 2C and F. The measured transverse and axial uniformity were 0.036 and 0.025, which was found to be the acceptable limit and is summarized in Table 5 and Fig. 2D . The alignment of the PET-CT registration was measured in the X, Y, and Z directions. The measured values are within the permissible limits at –0.251, –0.262, and 0.421 mm. It is summarized in Table 5 and Fig. 2E .

### uMIvista and Equivalent PET-CT Scanner

The evaluated NEMA NU2–2018 performance parameters of the uMIvista PET-CT system were compared with those of an equivalent PET-CT system. The comparison was performed with uMI550 PET-CT, GE Discovery MI and IQ PET-CTs, and Siemens Biograph Vision 600 PET-CT (as shown in Table 6 ).

**Table 6:** Comparison of NEMA technical parameters for PET scanners from different manufacturers

Model	UIH uMIvista	UIH uMI550	UIH uMI550	Siemens Biograph Vision 600	GE Discovery IQ	GE Discovery MI
Authors	Present study	Singh et al (2023) 4	Chen et al (2020) 7	Reddin et al (2018) 10	Reynés-Llompart et al (2017) 11	Pan et al (2019) 12
Manufacturer	United Imaging Healthcare	United Imaging Healthcare	United Imaging Healthcare	Siemens Healthineer	GE Healthcare	GE Healthcare
CT component	160 slices	80 slices	80 slices	128 slices	16 slices	128 slices
Specifications	NEMA NU2–2018	NEMA NU2–2018	NEMA NU2–2018	NEMA NU2–2018	NEMA NU2–2012	NEMA NU2–2012
AFOV (cm)	24	24	24	26.3	25	25
Crystal	LYSO	LYSO	LYSO	LSO	BGO	LYSO
Crystal dimension (mm)	2.76 × 2.76 × 16.3	2.76 × 2.76 × 16.3	2.76 × 2.76 × 16.3	3.2 × 3.2 × 20	6.3 × 6.3 × 30	4 × 4 × 25
Detector	SiPM	SiPM	SiPM	SiPM	PMT	SiPM
Sensitivity (cps/kBq)						
Center	12.05	10.35	10.24	15.1	20.8	20.81
10 cm	11.82	9.74	10.32	15.6	20.4	20.21
Spatial resolution						
Tan/rad/axial @ 1 cm	3.01/2.95/2.93	3/3/2.7	2.9/3/3	3.7/3.5/3.6	4.7/4.2/4.8	4.3/4.3/5
Tan/rad/axial @ 10 cm	3.17/3.42/3.05	3/3.4/3	3/3.3/3	3.9/4.6/4.3	5.1/5.6/4.8	4.6/5.5/6.5
Tan/rad/axial @ 20 cm	3.65/4.61/3.17	3.6/4.6/3.1	4/4.1/3.1	3.5/5.8/4.4	5.5/8.5/4.8	5/7.4/6.6
Image quality (%)						
10 mm (CR/BV)	54.6/6.2	50.5/6	46.5/6.4	86.8/6	18/4.4	46/9.3
13 mm (CR/BV)	60.6/5.1	62.3/4.8	76.2/5.5	72.2/5	37/4	54/7.1
17 mm (CR/BV)	66.5/4.0	74.7/3.9	79.3/4.7	85/3.9	59/3.6	66/5.4
22 mm (CR/BV)	74.7/3.2	81.6/3.3	82.8/4	89.8/3.3	70/3.4	71/4.4
28 mm (CR/BV)	85.7/2.7	82/2.7	82.1/3.3	87.4/3.0	61/3.3	85/3.8
37 mm (CR/BV)	88.5/2.3	84.5/2.2	83.9/2.5	89.6/2.2	64/3.5	89/3.5
Mean lung relative error	2.9	5.6	3.1	3.5	26	5.9
Count rate						
Scatter fraction (%)	37.66	38.5	36.65	39	36.2	40.2
Peak NECR (kcps)	452.98 (kcps) @ 29.95 (kBq/cc)	130.05 (kcps) @ 19.28 (kBq/cc)	124.4 kcps @ 18.85 kBq/mL	296 kcps @ 30.9 kBq/mL	123.6 kcps @ 9.1 kBq/mL	266.3 kcps @ 20.8 kBq/mL
Timing resolution (ps)	302	372	372	215	Non-TOF	381.7

Abbreviations: AFOV, axial field of view; BGO, bismuth germanate; cps/kBq, count per second per kilobecquerel; CR/BV, contrast recovery/background variability; LYSO; lutetium yttrium orthosilicate; NECR, noise-equivalent count rate; NEMA, National Electrical Manufacturer Association; PET, positron emission tomography; PMT, photomultiplier tube; ps, picosecond; SiPM, silicon photomultiplier; TOF, time-of-flight.

## Discussion

The uMIvista is the latest model of a digital PET-CT scanner. Post-installation technical performance evaluation was performed against NEMA NU2–2018 guidelines. 6 7 8 10 The collected NEMA NU2–2018 parameters were compared with existing equivalent PET-CT scanners for performance evaluation and are summarized in Table 6 . The compared PET-CT scanners had a similar physical PET field of view. The uMIvista PET-CT has a 24 cm wide PET field of view, while the uMI550 has 24 cm, the Discovery MI and IQ have 25 cm, and the Siemens Biograph Vision 600 has 26 cm. 4 7 10 11 12

## NEMA NU2–2018 Performance Parameters

The measured spatial resolutions in all three directions (10, 100, and 200 mm) of uMIvista were similar to those of Singh et al 4 and Chen et al 7 who studied uMI550 PET-CT scanners. This was because the physical and technical parameters of both PET-CT scanner models are similar, such as: crystal size, crystal type, detector type, PET ring diameter, and same manufacturer, as described in Table 6 . When compared with GE Discovery MI and IQ-PET-CT systems reported by Llompart et al 11 and Pan et al, 12 the measured spatial resolution of the uMIvista PET-CT system was assessed to be much higher. Similar higher spatial resolution compared with the Siemens Biograph PET-CT system was reported by Reddin et al. 10 This difference was measured with the uMIvista PET-CT system due to the crystal size. 10 11 12

The sensitivity of the uMIvista PET-CT system was found to be higher than the uMI550 PET-CT system reported by Singh et al 4 and Chen et al. 7 The sensitivity of the Discovery MI and IQ systems was highest (14). This significant difference was observed due to the thickness of the crystal. Thicker crystals achieve higher sensitivity. The crystal thickness of the uMIvista and uMI550 systems is thinner than the Siemens Biograph Vision and GE Discovery MI and IQ systems. This results in the highest sensitivity of Discovery IQ and MI, followed by Biography Vision, uMIvista, and uMI550. 4 7 10 11 12 The measured scatter fraction was 39.15% at maximum NECR and 37.66% at low activity. The measured fraction of scatter was consistent with the rest of the PET-CT systems, with minor deviations, as shown in Table 6 . The standard values for the scatter fraction range from 35 to 40%. It was observed that uMIvista and other PET-CT systems had shown similar results. The peak true count rate and NECR measurements of uMIvista were found to be higher than those of Singh et al 4 and Chen et al 7 who examined uMI550 system. In addition, it was also higher than the GE Discovery MI and IQ systems and the Siemens Biograph Vision 600. The measured TOF timing resolution of the uMIvista PET-CT system was 302.294 ps, reportedly superior to uMI550 studied by Singh et al 4 and Chen et al 7 as well as to GE. The measured TOF time resolution of the uMIvista PET-CT system was 302.294 ps and was reportedly superior to that of Singh et al 4 and Chen et al 7 who examined the uMI550 and the GE Discovery MI PET-CT system, while the TOF time resolution of the Siemens Biograph Vision 600 is higher than that of the uMIvista PET-CT. Image quality on the NEMA body phantom in terms of CRC, BV, and relative lung error was measured and observed to be consistent with other digital PET-CT systems (see Table 6 ) and the analog Discovery IQ system. This was due to the superior performance of SiPM detectors over PMT, as summarized in Table 6 .

## Conclusion

The NEMA NU2–2018 performances of this TOF-integrated digital PET-CT system are observed to be superior for various parameters.
